# Supervised machine learning and genotype by trait biplot as promising approaches for selection of phytochemically enriched *Rhus coriaria* genotypes

**DOI:** 10.1016/j.heliyon.2024.e41548

**Published:** 2024-12-29

**Authors:** Hamid Hatami Maleki, Reza Darvishzadeh, Ahmad Alijanpour, Yousef Seyfari

**Affiliations:** a Department of Plant Production and Genetics, Faculty of Agriculture, University of Maragheh, Maragheh, Iran; b Department of Plant Production and Genetics, Faculty of Agriculture, Urmia University, Urmia, Iran; c Department of Forestry, Faculty of Agriculture and Natural Resources, Urmia University, Urmia, Iran; d Faculty of Engineering, University of Maragheh, Maragheh, Iran

**Keywords:** Artificial intelligence, ISSR primer, Phytochemical marker, Sumac

## Abstract

Sumac is considered as a medicinal and industrial plant. Climate change threats natural ecosystems and hence, evaluation of sumac's genetic diversity, identification of superior genotypes, and conservation of such materials is important. In this study, 5 wild populations of sumac were investigated. Fruits of 75 sumac genotypes (15 genotype per population) were analyzed using HPLC-LC/MS-MS method. Likewise, genomic DNA of 75 genotypes were fingerprinted using 18 ISSR primers. Analysis of variance revealed significant genetic variability among studied populations of sumac considering malic acid, malic acid hexoside 2.71, malic acid hexoside 6.11, coumaric acid, ellagic acid11.49. Malic acid was identified as phytochemical marker in sumac fruit which can be implemented for screening sumac genotypes even from the same population. Genotype by trait analysis revealed V6, V10, D10, D14, A1, A14, K3, K15, N10, and N11 as top-performing genotypes (winners) which possessed the majority of phytochemical constituents in highest value. Here, the identified phytochemically superior sumac group was effectively distinguished from the inferior sumac group using ISSRs information via supervised machine learning. By using 13 feature selection algorithms, ISSR loci (U823) L1, (U835) L1, (U801) L1, (U816) L2, (U816) L4, (U835) L4, (U854) L1, and (U835) L9 were identified as functional markers which could predict phytochemical response of sumac germplasm. In conclusion, there is vast range of phytochemically divergent sumac genotypes in its natural habitats that could effectively recognized in any season by merging artificial intelligence with genomic information.

## Highlights

• Genetic variability in habitats of sumac can be clarified by phytochemical records.
• Malic acid as phytochemical marker in sumac fruit could distinguish sumac genotypes.
• ML through DNA markers facilitates detection of phytochemically superior genotypes.

## 1 Introduction

*Rhus coriaria* L. is one of the medicinal plant species in family *Anacardiaceae* [1] is native to southern Europe and western Asia. It has chromosomal formula of 2n = 2x = 30 with narrow knowledge about its genome sequence. In Iran, sumac is distributed mostly in the foothills of numerous parts of northern regions such as Azerbyjan, Qazvin, Albourz and Khorasan provinces. Regarding its anti-cancer [2], anti-diabetic [3], anti-bacterial [4], and anti-fungi [5] attributes, sumac has many applications in conventional health care. Also, dried fruits of sumac which changed to a dark red powder, with an acidic and astringent taste, is often used as a spice in several Mediterranean and Middle Eastern countries such as Lebanon, Syria, Jordan, Turkey, and Iran [6]. Until now, over 200 compounds have been identified from the *Rhus coriaria* which most of them are physiologically active [7]. These chemical constituents can be assigned to various classes of the hydrolysable tannins, phenolic acids, conjugated phenolic acids, anthocyanins, flavonoids, organic acids, coumarins, xanthones, terpenoids, steroids, essential oils, and other groups of constituents.

Biodiversity measurement is an important factor for evaluating ecosystems because ecosystems with higher biodiversity have more ecological stability and production. Likewise, accurate assessment of the amount and the distribution of genetic diversity in rare and endangered species is of particular importance to develop a strategy for protection and use of genetic resources. In this regard, several phytochemical investigations [8,9] about the constituents of *Rhus coriaria* have established and resulted that its fruit possesses the majority of the chemical constituents. Moreover, phytochemical variability relevant to sumac's fruits sampled from different geographical regions was proved using GC-mass, HPLC, and their derived technologies to analyze sumac fruit [6,7,10,11]. Generally, using HPLC-MS and Ethanolic extract the existence of compounds such as phenolics, flavonoids, anthocyanins, tannins, and organic acids were proved in sumac fruits [12,13]. Also, by GC-MS analysis the presence of volatiles, and fatty acids were reported [14,15].

Besides emphasizing on phytochemical properties of sumac as a medicinal plant [16], it is vital to recognize superior phytochemically enriched sumac genotypes within its natural habitats as well as over habitats. Likewise, determination of discriminative phytochemical constituents of sumac which results in differentiation of sumac's genotypes from each other is unavoidable. According to literature, Farag et al. [10] analyzed fruit's volatile components of three sumac accessions from Palestine, Jordan and Egypt and finally reported the existence of 74 volatile components. Also, Oscan et al. [6] studied 15 sumac genotypes sampled from several regions of Kahramanmaras province of Turkey and found sumac fruit as a good medicinal and nutritional source with valuable phytochemical components including phenolics, anthocyanins, organic acids and carbohydrates. In another research [11], phytochemical properties of sumac fruits belonging to five Sicilian accessions were inspected and isoquercitrin and gallic acid were determined as two main constituents of its fruit. Detailed focusing on studies undertaken in sumac shows that majority of aforementioned reports tended to reveal the existence of variability and possibility for selection of interested chemotype. As inferred, none of them tried to introduce phytochemical biomarker for sumac which could effectively distinguish its samples. Paralleled with phytochemical analysis of sumac fruit, application of DNA markers [17,18] also addressed potentially genetic variability in sumac germplasm and the efficacy of DNA markers in distinguishing sumac populations. For instance, Sutyemez et al. [17] studied genetic variability among 24 sumac genotypes using 17 SRAP and 12 ISSR primers and by detecting 133 loci emphasized the efficacy of these marker systems in evaluation of sumac germplasm. Kılıncer et al. [18] had been used SSR markers designed based on transcriptomics data and reported two sub-populations in sumac from Anatolia. But from a breeder's point of view, there is gaps about utilization of DNA markers as predictive tool in identification of genotypes and speeding up the breeding programs through its usage in each plant growth stage. Recently, advent of artificial intelligence as a powerful computational method and other robust graphical statistical analysis such as GGE biplot could led to successful harnessing in plant germplasm evaluation. Machine Learning (ML) as an area of artificial intelligence [19] with two types of supervised and unsupervised learning methods translate the features in the training data into pattern, and clustering/prediction of the labels. Unsupervised ML methods are used when the labels on the input data are unknown; these methods learn only from patterns in the features of the input data. In supervised methods, on the other hand, labeled features are trained to predict the class labels based on training examples. There are several reports that used ML algorithms to classify and predict plant germplasm such as olive [20], common bean [21], potato [22], grape [23] using DNA marker data. In addition, in genotype-trait (GT) biplot as applicable subject of GGE analysis the genotypes are consider as lines and traits as testers and finally select various attributes with high performance and success. There are some reports about successful application of GT analysis in crops including maize [24], and wheat [25].

By concentrating on sumac's superior genotypes in each habitat, it is possible to enrich sumac germplasm through intelligent germplasm conservation. Hence, this study aimed to evaluate genetic variability among 75 sumac samples from five regions of Iran using HPLC-LC/MS-MS analysis and ISSR marker assay. In the following, phytochemically winner genotypes in each population were selected by genotype-trait analysis and also ISSR loci which could predict sumac enriched genotypes were introduced by supervised machine learning method.

## 2 Materials and methods

### 2.1 Plant material and phytochemical constituents

**Fig. 1:**
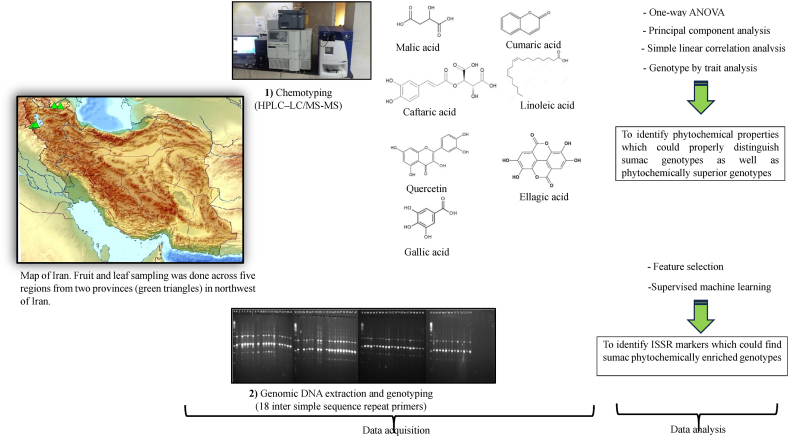
Schematic view of sumac's sampling regions, phytochemical and genetic data recording, and analysis procedure.

**Fig. 2:**
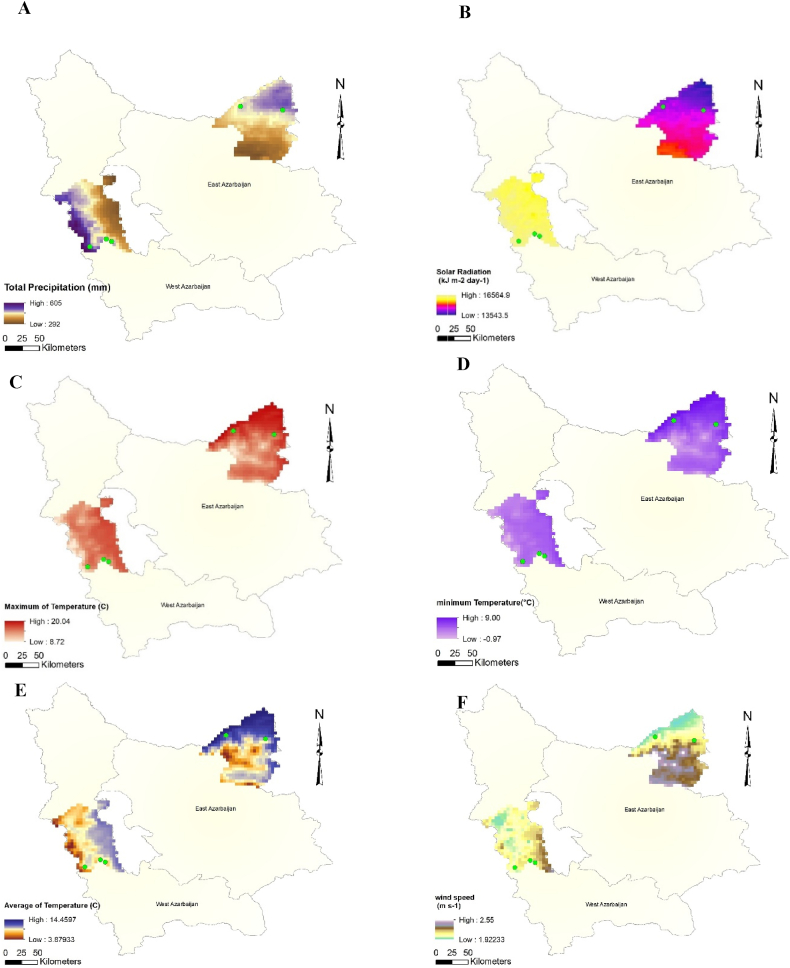
Coordinates of sampling regions located in two provinces (West Azerbaijan and East Azerbaijan). In each province, sampling regions had been shown with green color. Graphs A to F depicted total precipitation, solar radiation, maximum temperature, minimum temperature, average temperature, and wind speed.

In the present study, 75 sumac genotypes from five various regions ("Kachleh", "DarrehKhan" "DarrehNezh", "Aghberaz", and "Vinagh") had been studied (Table 1, Fig. 1). These regions belong to two provinces comprising East Azerbaijan, and West Azerbaijan of Iran (Table 1, Fig. 1). For each region, precipitation (Fig. 2A), solar radiation (Fig. 2B), maximum temperature (Fig. 2C), minimum temperature (Fig. 2D), the parameters of average temperature (Fig. 2E), and wind speed (Fig. 2F) with an accuracy of 2.5 min were prepared from the global climate and meteorological data database that covers from 1970 to 2020. Afterward, the polygon view of the regions where sampling was done prepared in ArcGIS version 10.8. In the following, mature fruits of 15 genotypes for each population were collected. For phytochemical analysis (Fig. 1), after drying fruits, epicarps of fruits were separated from kernels and ground to powder by the household mill. For extraction of phenolic compounds 10 ml Methanol (HPLC grade) (80 % v/v) added to grounded fruit epicarps (0.5 gr) and sonicated for 45 min at room temperature. The mixture was centrifuged for 15 min at 3000 g at room temperature and then filtered through a 0.2 μm syringe filter (PALL, USA) and stored at 20 °C until analysis. HPLC–LC/MS-MS analysis was performed using an Agilent ZORBAX SB-C18 (4.6 × 150 mm, 5 μm). The mobile phase was composed of water (A, 0.1 % formic acid) in methanol (B, 0.1 % formic acid), the gradient program of which was 0–1.00 min 55 % A and 45 % B, 1.01–20.00 min 100 % B and finally 20.01–23.00 55 % A and 45 % B. The flow rate of the mobile phase was 0.25 mL/min, and the column temperature was set to 30 °C. The injection volume was 10 μL. The optimum ESI parameters were determined as 2.40 mTorr CID gas pressure, 5000 V ESI needle voltage, 600 V ESI shield voltage, 300 °C drying gas temperature, 50 °C API housing temperature, 55 psi Nebulizer gas pressure and 40.00 psi drying gas pressure. Mass Lynx software, version 4.1, was used for instrument control and data acquisition. The analysis was performed in positive and negative ion mode.

**Table 1:** genotype code, name and geographical coordinates of the collected sumac populations.

Genotype code	Region	Province	Latitude	Longitude	Height (m)	Slope
V1-V15	Vinagh	East Azerbaijan	39.03	46.83	860	60
A1-A15	Aghberaz		38.98	47.38	1190	45
D1-D15	DarrehKhan	West Azerbaijan	37.30	45.08	1533	10
K1-K15	Kachleh		37.20	44.87	1727	55
N1-N15	DarrehNezh		37.27	45.15	1623	15

### 2.2 ISSR assay

Genomic DNA was extracted from leaf samples of each sumac genotype with dBIOZOL Genomic DNA Extraction Reagent. A suite of 18 ISSR primers (Table 2) were implemented for fingerprinting of sumac genotypes. The polymerase chain reaction was done as following program: an initial denaturation at 94 °C for 4 m, followed by 36 cycles of 94 °C for 60 s, annealing temperature depending on primers composition (52–60 °C, Table 1) for 45 s, and 72 °C for 2 m, with a final extension at 72 °C for 10 m. The amplified DNA fragments were separated through agarose gel (%1.8) electrophoresis.

**Table 2:** Name, sequence, and molecular characteristics related to utilized ISSR primers.

Primer	sequence (5′ to 3′)	Annealing temperature	Amplified loci	PIC^a^
UBC811	(GA)8C	49	6	0.36
UBC823	(TC)8C	48	3	0.30
UBC826	(AC)8C	50	5	0.40
UBC835	(AG)8 YC	52	10	0.36
UBC842	(GA)8CTG	50	8	0.34
UBC854	(TC)8RG	44	4	0.33
UBC856	(AC)8 YA	44	6	0.30
UBC834	(GA)8 YT	50	7	0.32
UBC841	(GA)8CTC	52	12	0.46
UBC809	(AG)8G	46	6	0.23
UBC810	(GA)8T	38	15	0.34
UBC857	(AC)8 YG	40	9	0.39
UBC867	(GGC)8	39	12	0.32
UBC866	(CTC)6	53	4	0.37
UBC864	(ATG)6	42	6	0.41
UBC801	(AT)8T	50	7	0.32
UBC890	VHV (GT)7	52	4	0.39
UBC816	(GA)8T	50	8	0.41

a Polymorphism information content.

### 2.3 Phytochemical data analysis

After collecting phytochemical data, the presence of outliers was checked using Minitab 15.0 software. Analysis of variance and Duncan's mean comparison test was done by considering population as treatment and samples within each population as replications through command "aov" and " duncan.test" in package "agrioclae". Simple Pearson correlation coefficient among recorded phytochemical constituents was calculated by "corrplot" package and then principal component analysis was exerted by using "factoextra" package. The software R was implemented for the mentioned statistical analysis. With the aim of genotype by trait analysis, the two-dimensional data matrix (sumac genotypes-phytochemical constituents) was constructed and then implemented via the GGE-biplot [26]. This model graphically represents the pattern of GT interaction using the following formula:

$$\frac{X_{ij}-\mu_{j}}{S_{j}}=\sum_{n=1}^{2}\alpha_{n}\beta_{in}\gamma_{jn}+E_{ij}$$

In the formula provided: X_ij_ represents the genotype i for character j, μ_j_ is the mean of genotypes for character j, S_j_ is the root square of variance in character j across genotypes, α_n_ is the singular value, β_in_ and η_jn_ are PC-values of genotypes and characters, E_ij_ is the residual magnitude of the model. To achieve symmetric scaling in the values of both genotypes and traits, the singular value α_n_ needs to be adjusted via absorption of their vectors (β_in_ and *γ*_jn_). This adjustment helps in obtaining a balanced representation of treatments and traits in the analysis; $\beta_{in}^{*}=\sqrt{\lambda_{n}}\xi_{in}$ and $\gamma_{jn}^{*}=\sqrt{\alpha_{n}}\gamma$. The GT interaction biplot graphs are created by plotting the symmetrical values of the genotypes and traits. In these graphs, genotypes and traits are shown by a unique marker.

### 2.4 ISSR scores and supervised machine learning

ISSR amplified fragments were scored as zero (absent) and 1 (present) to produce a binary data matrix. Then, polymorphism information content as criteria for identification of primer power in evaluation of germplasm's genetic variability was calculated as described [27] formula of PIC = 1 - [f^2^ + (1 - f)^2^], where f is the marker frequency in the data set.

Further analysis by supervised machine learning was done through assuming identified phytochemically enriched sumac genotypes (V6, V10, D10, D14, A1, A14, K3, K15, N10, and N11) as a separate group (superior group) and other studied sumac genotypes in another group (inferior group) in MATLAB software. To select the most important and discriminative features (ISSR loci) which could predict the mentioned classification, original data accompanied with 36 data collections produced by 36 feature selection algorithms imposed to 16 classification algorithms (Supplementary Table 1). The combination of feature selection algorithms × classification algorithms (Supplementary Table 1) with average accuracy higher than 0.85 was selected as suitable one in separation of the superior group from the inferior group. In this study, a variety of Correlation-based Feature Selection methods including Filtered Attribute Evaluation, Filtered Subset Evaluation, Chi-Squared, Information Gain, Gain Ratio, and Probabilistic Significance were used. The details of these algorithms are presented in Table 1. For methods 1 to 6, Correlation-based Feature Subset Selection was used, where subsets of features that are highly correlated with the class but have low intercorrelation among themselves were preferred. However, the search method for subset selection varied for each correlation-based method. The thresholds used for methods 6, 7, 10, 11, 12, and 13 are also provided in Table 3.

**Table 3:** List of feature selection algorithm and correspondence method.

No.	Feature selection algorithm/search method	Threshold	ISSR loci as significant features^a^
1	Correlation-based Feature Subset Selection	–	(U823)L1, (U835)L1, (U835)L4, (U854)L1, (U801)L1, (U816)L2 (U816)L4
	Search Method: BestFirst		
2	Correlation-based Feature Subset Selection	–	(U823)L1, (U835)L1, (U801)L1, (U816)L2, (U816)L4
	Search Method: incremental wrapper feature subset selection with Naive Bayes		
3	Correlation-based Feature Subset Selection	–	(U823)L1, (U835)L1, (U835)L4, (U854)L1, (U801)L1, (U816)L2, (U816)L4
	Search Method: Linear Forward Selection		
4	Correlation-based Feature Subset Selection	–	(U823)L1, (U835)L1, (U835)L4, (U854)L1, (U801)L1, (U816)L2, (U816)L4
	Search Method: Scatter Search		
5	Correlation-based Feature Subset Selection	–	(U823)L1, (U835)L1, (U835)L4, (U854)L1, (U801)L1, (U816)L2, (U816)L4
	Search Method: Linear Forward Selection (subset size selection)		
6	Correlation-based Feature for Subset Selection	>0.5	(U835)L1, (U816)L2, (U835)L4, (U801)L1, (U816)L4, (U823)L1, (U854)L1, (U835)L9
	Search Method: Random		
7	Filtered Attribute Eval	>0	(U816)L2, (U835)L1, (U835)L4, (U816)L4, (U801)L1, (U823)L1, (U854)L1, (U835)L9
8	Filtered Subset Eval	–	(U823)L1, (U835)L1, (U835)L4, (U854)L1, (U801)L1, (U816)L2, (U816)L4
	Search Method: Greedy Stepwise		
9	Filtered Subset Eval	–	(U823)L1, (U835)L1, (U835)L4, (U854)L1, (U801)L1, (U816)L2, (U816)L4
	Search Method: BestFirst		
10	Chi Squared	>0	(U816)L2, (U835)L1, (U816)L4, (U835)L4, (U801)L1, (U823)L1, (U854)L1, (U835)L9
11	Information Gain	>0	(U816)L2, (U835)L1, (U835)L4, (U816)L4, (U801)L1, (U823)L1, (U854)L1, (U835)L9
12	Gain Ratio	>0	(U835)L1, (U816)L2, (U835)L4, (U801)L1, (U823)L1, (U854)L1, (U835)L9, (U816)L4
13	Probabilistic Significance	>0	(U816)L2, (U835)L1, (U816)L4, (U835)L4, (U801)L1, (U823)L1, (U854)L1, (U835)L9

a Meaningful features that identified via each feature selection algorithm.

## 3 Results

### 3.1 Phytochemical properties of studied sumac populations

**Fig. 3:**
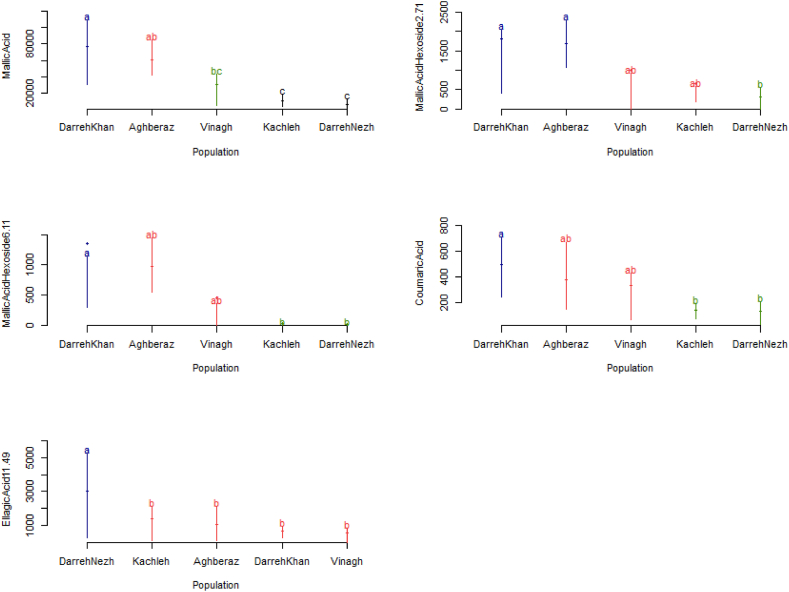
Mean comparison among populations of sumac using Duncan multiple range test. In each case, populations without any significant difference showed by the same letter.

A varied range of several phytochemical ingredients including gallic acid 6.5, gallic acid 8.7, quercetin, malic acid, malic acid hexoside 2.71, malic acid hexoside 6.11, coumaric acid, coumaric acid 8.9, caftaric acid, linoleic acid, linoleic acid5, ellagic acid11.49, and ellagic acid13.97 were recorded in fruits of 75 sumac genotypes. Analysis of variance (Table 4) revealed existence of significant genetic variability among studied sumac populations from Iran considering malic acid, malic acid hexoside 2.71, malic acid hexoside 6.11, coumaric acid, ellagic acid11.49. In the present study, non-significant effects for the rest of measured phytochemical constituents were detected (Table 4). In order to find which differences, lead to discrimination between studied population, the DNMRT test has been applied (Fig. 3) and it showed that "Aghberaz" population (East Azerbaijan province) and "DarrehKhan" population (West Azerbaijan province) have no significant differences together in the case of significant phytochemical constituents except of ellagic acid11.49. Albeit, "DarrehKhan" had the maximum values of malic acid and coumaric acid but the maximum values of malic acid hexoside 2.71 and malic acid hexoside 6.11 were detected for " Aghberaz" population (Fig. 3). Result concerned with EllagicAcid11.49 showed that it differed among studied populations and sumac genotypes from "DarrehNezh" (West Azerbaijan province) population have the maximum value of it (Fig. 3).

**Table 4:** One way analysis of variance for phytochemical constituents of sumac's fruit.

		Mean of square						
Source of variation	Degree of freedom	gallic acid 6.5	gallic acid 8.7	Quercetin	malic acid	malic acid hexoside2.71	malic acid hexoside6.11	coumaric acid
Between population	4	267075ns	408922 ns	3034 ns	1.45E+10∗∗	6211220∗∗	5076174∗∗	371797∗∗
Within population	70	366921	503444	5802	1.33E+09	1579125	920738	72326
CV%	–	121.92	102.42	145.44	98.43	115.46	168.53	90.23

Source of variation	Degree of freedom	Mean of square						
		coumaric acid8.9	Caftaric acid	linoleic acid	linoleic acid5	ellagic acid11.49	ellagic acid13.97	
Between population	4	8944 ns	188.3 ns	32.19 ns	37031 ns	15150211∗∗	425792 ns	
Within population	70	7891	97	23.08	19870	2212658	596657	
CV%	–	99.12	142.32	105.35	152.37	110.16	113.41	

^ns^and ∗∗ are non-significant and significant at 1 % probability level respectively.

**Fig. 4:**
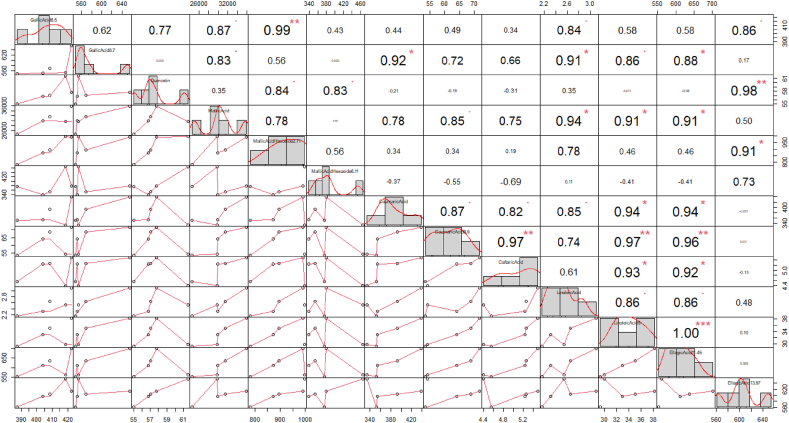
Simple Pearson correlation between measured phytochemical constituents.

Likewise, Pearson's correlation coefficient among studied phytochemical traits (Fig. 4) showed that the majority of calculated correlation coefficients are positive which depicts alignment changes in phytochemical characteristics of sumac' fruit. As shown in Fig. 4, the correlation values varied between zero (malic acid hexoside 6.11 with gallic acid 8.7) and one (gallic acid 8.7 with ellagic acid11.49). Albeit, malic acid hexoside 2.71 with gallic acid 8.7 also had the near to maximum value of correlation (r = 0.99) (Fig. 4). Here, positive significant linear correlation was also detected between malic acid with phytochemical constituents such as linoleic acid, linoleic acid5, and ellagic acid11.49 at 5 % of probability level (Fig. 4). Also, coumaric acid 8.9 as another substantial constituent of sumac fruit had positive significant relation with caftaric acid, linoleic acid5, and ellagic acid11.49 at 1 % of probability level. Results have proved the existence of a parallel significant correlation between gallic acid 8.7 with constituents including coumaric acid, linoleic acid, linoleic acid5, and ellagicAcid11.49 (Fig. 4).

**Fig. 5:**
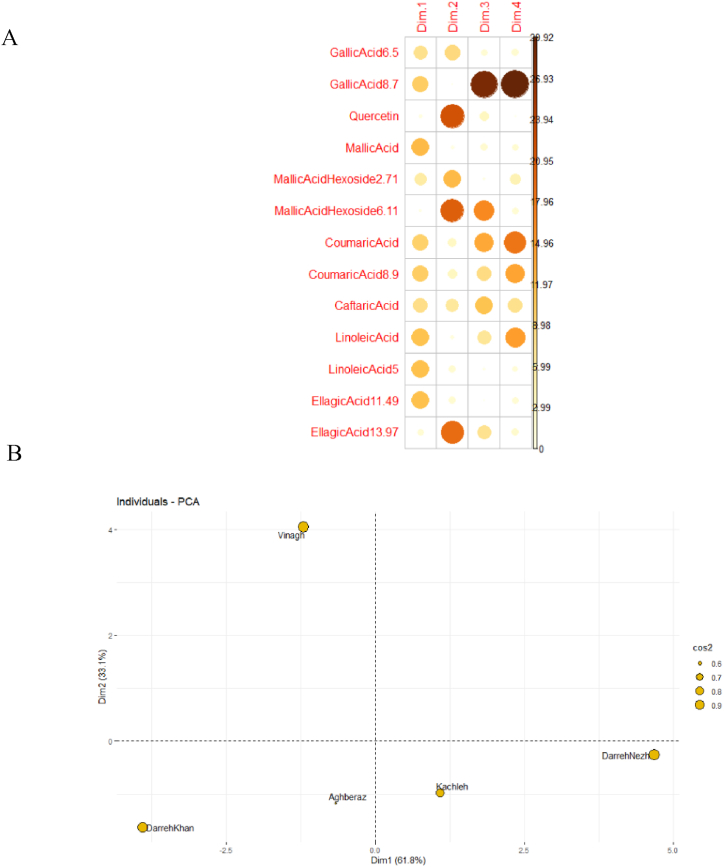
PCA analysis of sumac germplasm considering phytochemical constituents of fruit, A) resulted four PCs which each attribute loading coefficient showed by color circles and intensity of color manifested its importance, B) classify individuals through Cos2 value which indicates quality of representation of individuals on factor map.

Principal component analysis as robust multivariate statistical analysis could be applied to reduce dimensions of data and also enable scientists to make exact decision. In the PCA analysis, the studied data is summarized in some significant components (PCs) and afterward all of the analysis and decisions are made by newly introduced PCs. Here, by applying PCA analysis on 13 phytochemical constituents of sumac fruit, 4 PCs were identified which the first 2 PCs (Dim1 and Dim2) had eigenvalues >1 and explained 94.82 % of the total variation (Fig. 4). In the first PC (dimension 1), all of the measured phytochemical characters except for quercetin, malic acid hexoside 2.71, malic acid hexoside 6.11, and ellagic acid13.97 had important roles while in the second (dimension 2) gallic acid 6.5, quercetin, malic acid hexoside 2.71, malic acid hexoside 6.11, coumaric acid, coumaric acid 8.9, caftaric acid, and ellagic acid13.97 had remarkable loading coefficients (Fig. 5A). As visualized in Fig. 5A, the loading coefficients related to fruit phytochemical characters are not so dispersed in PC1 whilst they are varied in PC2 and so phytochemical constituents such as quercetin, malic acid hexoside 6.11, and ellagic acid13.97 have highest values of loading coefficients which implies their importance in PC2. Since the first two factors explained the majority of variation existed in phytochemical data so the bi-plot involves these two PCs (Fig. 5B), regarding the contribution of PCs in each population, was applied for classifying studied sumac populations. In this term, the size of circle related to each population indicates the proportion of variability, which is explained by both PCs and the large one indicates reliability of population position in the bi-plot. As shown in Fig. 5B, except for "Aghberaz" (East Azerbaijan) population, the two PCs have greater contribution (Cos2 = 0.9) in explaining their fruit's phytochemical constituents of studied sumac populations. As inferred from bi-plot, sumac populations from West Azerbaijan province including "DarrehKhan", "DarrehNezh", and "Kachleh" populations with PC1 (Dim1) and PC2 (Dim2) values near together could be assign in same group (Fig. 5B). Notably, the "Vinagh" population (East Azerbaijan province) was distinguished from other ones even population "Aghberaz" regarding Dim1 and Dim2 coordinates.

### 3.2 Superior genotypes selection using phytochemical markers

**Fig. 6:**
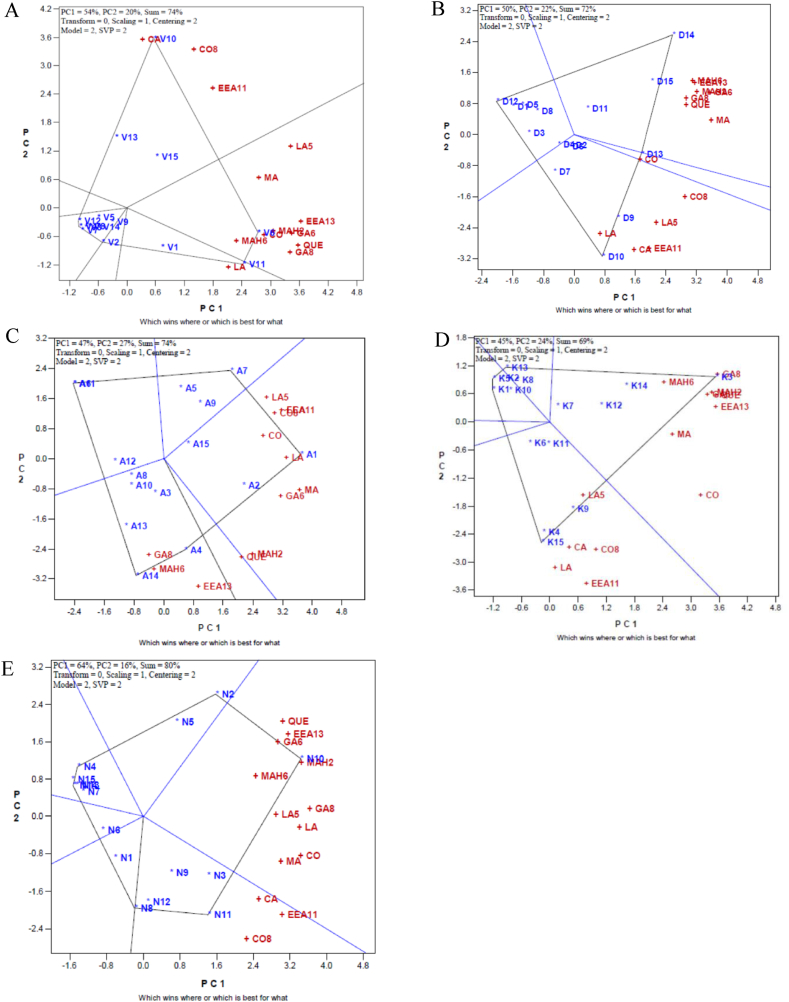
Biplot indicating which genotypes were the wining ones for which phytochemical constituent of sumac. A) Vinagh, B) DarreKhan C) Aghberaz, D) Kachleh, E) DarrehNezh.

**Fig. 7:**
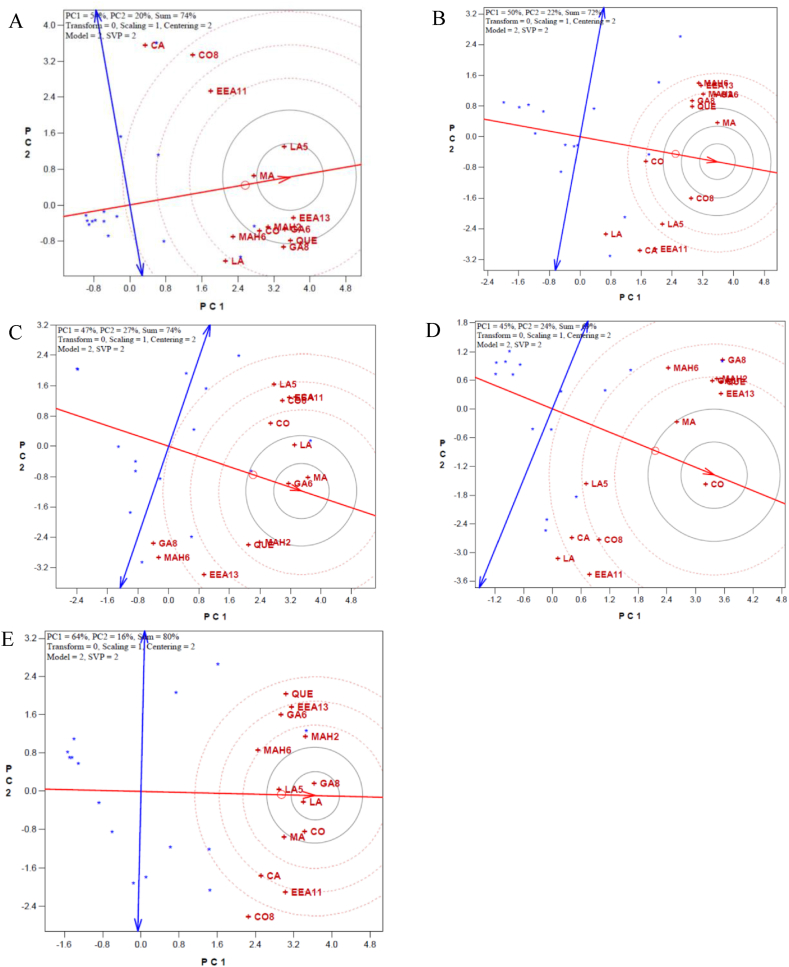
Biplot indicating position of ideal constituent regarding phytochemical constituents of sumac. A) Vinagh, B) DarreKhan C) Aghberaz, D) Kachleh, E) DarrehNezh.

In the following, to find out phytochemically enriched sumac genotypes from each population the genotype by trait (GT) biplot analysis was done for each habitat of "Vinagh" (Fig. 6A), "DarreKhan" (Fig. 6B), "Aghberaz" (Fig. 6C), "Kachleh" (Fig. 6D) and "DarrehNezh" (Fig. 6E) separately. The first and second PCs derived from the model explained more than 70 % of data variability in all studied habitats. It is resulted (Fig. 6) that which genotypes excelled for which phytochemical character in each habitat. In this way, genotypes V10 and V6; from "Vinagh" population, genotypes D10 and D14; from "DarrehKhan" population, genotypes A1 and A14; from "Aghberaz" population, genotypes K3 and K15; from "Kachleh" population, and genotypes N10 and N11, from "DarrehNezh" population were selected as the top-performing genotypes (winners) which possessed highest values of measured constituent. About measured phytochemical traits, it is beneficial to identify an ideal trait that enable to differentiate the sumac genotypes from each other (Fig. 7). Accordingly, as illustrated by GT analysis (Fig. 7), the arrow in the circles’ middle has the largest vector of the phytochemical characters with positive projections onto the average of coordinate axis of traits. A phytochemical character is more favorable if it is closer to the location of the special trait, therefore, MA (malic acid) following to LA5 (linoleic acid5) were identified as favorable trait in "Vinagh" population (Fig. 7A). Regarding obtained results, in the "DarrehKhan" population (Fig. 7B), MA (malic acid) accompanied by CO8 (coumaric acid8.9), in the "Aghberaz" population (Fig. 7C), MA (malic acid) and GA6 (gallic acid 6.5) and in the "Kachleh" population (Fig. 7D), MA followed by CO (coumaric acid) were identified as favorable phytochemical traits. Genotype by trait analysis (Fig. 7E) showed importance of LA (LinoleicAcid) and GA8 (Gallic Acid 8.7) are favorable traits which could distinguish individuals in "DarrehNezh" population. Although, malic acid had also good rank "DarrehNezh" population.

### 3.3 ISSR markers utilized for genetic variability and supervised machine learning

Fingerprinting of sumac genotypes using ISSR primers detected 132 loci (Table 2) with a mean of 7.3 loci per allele. Among studied ISSR primers, minimum (0.23) and maximum (0.46) values of PIC were calculated for UBC809 and UBC841 respectively. In this study, 16 classification algorithms accompanied by 36 feature selection algorithms were applied on 132 loci produced through ISSR assay. Results of supervised machine learning (Table 3) showed that artificial intelligence could predict the phytochemically detected superior as well as inferior sumac groups in the exact manner. Among studied combinations of classification algorithm × feature selection algorithm, 13 combinations (Supplementary Table 1) were selected to possess an average accuracy of more than 0.85 (Table 3 and Supplementary Table 1). As shown in Supplementary Table 1, when no feature selection was applied in DNA marker data, none of classification algorithm could achieve predefined superior and inferior groups precisely. Aforementioned 13 feature selection algorithms (Table 3) jointly emphasized on five ISSR loci ((U823)L1, (U835)L1, (U801)L1, (U816)L2, (U816)L4) as functional loci for discrimination of the superior and the inferior genotypes from each other. However, all feature selection algorithms except for "Correlation-based Feature Selection methods with search method of Naive Bayes" also depicted the critical role of ISSR loci (U835)L4, (U854)L1, and (U835)L9 in predicting sumac groups.

## 4 Discussion

The current study showed a wide range of genetic variability among sumac populations collected from five regions of Iran regarding recorded phytochemical properties. All of phytochemical constituents which were detected in the present study were also reported by Batiha et al. [9] in sumac fruit. Such phytochemical variation had been also reported by Grassia et al. [28] among three sumac fruit samples and Mazzara et al. [11] among five sumac fruits samples belonging to sicilian sumac. In other study, paralleled with our findings, Ozcan et al. [6] had been shown remarkable genetic variability among 15 sumac genotypes which sampled from Kahramanmaras province of Turkey. Also, about volatile compound of sumac, Farag et al. [10] reported genetic variability among sumac genotypes sampled from three distinct regions including Palestine, Jordan, and Egypt. The major difference between present research with aforementioned studies, is inspection of large number of samples within each sumac population and so this is applicable to study both between and within population variability. As resulted by analysis of variance, the present sumac populations are differed phytochemically even with high value of coefficient of variation (CV%) so it could be inferred that between population differences is remarkable. Indeed, this is expected because according to Pant et al. [29] the meteorological attributes of studied sites are varied (Fig. 2). Particularly, even meteorological properties within each province are also varied (Fig. 2). In this study, high values of genetic variability were also detected within each population based on phytochemical measurements which is evidence for high values of CV%. Hence, there is significant within population genetic variability for sumac which could enhance genetic gain [30] and so, it is possible to achieve phytochemically enriched sumac genotypes by means of selection method.

Our results showed that malic acid and its derivatives accompanied with coumari acid, and ellagic acid are significantly varied among studied sumac populations. These ingredients are important, for instance coumaric acid [11] as a derivative of Hydroxycinnamic acids act as powerful antioxidants and protect biologically important molecules from oxidation. Likewise, ellagic acid [31] is a bioactive polyphenolic compound naturally occurring as secondary metabolite in many plant taxa. It has antioxidant, antimutagenic, and anticancer properties. Also, malic acid and it's derivatives, were recorded to be the most abundant organic acids in sumac [32]. So, it is concluded that present sumac germplasm as versatile and resourceful food have need more attention not only for germplasm conservation rather developing and introducing new chemotype. Inspection of merits of studied populations regarding aforementioned phytochemical ingredients showed "DarrehKhan" (malic acid, and coumaric acid), "DarrehNezh" (ellagic acid11.49), and "Aghberaz" (malic acid hexoside 2.71 and malic acid hexoside 6.11) contained the highest concentration of some phytochemicals and therefore, have potential to contribute to nutrition security distinguishable from other ones. Similarly, Morshedloo et al. (2022) as well as Sabaghnia et al. [33] reported divergence among Iranian sumac populations based on morphological and fatty acid profile, respectively.

In the present work, significant positive correlations were found among measured phytochemical constituents which prompt this opinion that the measured characters will allow direct and indirect selection of the evaluated genotypes for phytochemical quality improvement. As resulted, there was not any correlation between malic acid hexoside 6.11 with gallic acid 8.7 which is expected because malic acid hexoside 6.11 as derivative of malic acid belonged to organic acids while gallic acid 8.7 hydrolysable tannins [34]. Moreover, malic acid and linoleic acid had positive correlation which is predictable [9] in order to possessing to same class of compounds (organic acids). It is concluded that some organic acids for instance linoleic acid has positive correlation with ellagic acid as hydrolysable tannins so such compounds could indirectly be applied for increasing hydrolysable tannins. Beside phytochemical attributes of studied sumac populations, it is beneficial to classify and recognize heterotic groups in studied sumac populations as well. Here in, studied sumac populations was dispersed in space of bi-plot produced by PCA analysis. Interestingly, populations classification pursues their geographical distribution and populations from two provinces were separatable from each other. Empirically, by focusing at distant groups it will be facilitated to identify varied chemotype of sumac. Likewise, it is also possible to select parental genotype from distant groups and broaden transgressive segregants of sumac.

In the following, within population genetic variability was examined by GT biplot analysis. Recently, this method had been implemented in several field crops [35,36] but narrow studies have been reported in medicinal plants specially sumac. In this study, using GT biplot analysis genotypes V6 and V10 from "Vinagh" population, D10 and D14 from "DarrehKhan" population, A1 and A14 from "Aghberaz" population, K3 and K15 from "Kachleh" population, and N10 and N11 from "DarrehNezh" population were selected as winner sumac genotypes which has majority of phytochemical constituent in high level. As novelty of present study, the GT biplot analysis showed which sumac's phytochemical markers could effectively clarify within sumac population variability and distinguish sumac genotypes. Accordingly, malic acid was proved as remarkable phytochemical marker that could implemented for evaluation of genetic diversity within natural habitats of sumac including "Vinagh", "DarrehKhan", " Kachleh", and " Aghberaz", and "DarrehNezh" populations. From practical view, present research showed that genotypes including V6, V10, D10, D14, A1, A14, K3, K15, N10, and N11 are phytochemically superior genotypes among studied sumac germplasm from Iran and malic acid could be calculated as biochemical marker which has incredible role in distinguishing individuals within population of sumac. But, regarding to literature review [37], there are some limitations in application of such types of markers in evaluation of interested plant germplasm regarding their frequencies, late appearance, and environmental effects. While, DNA markers have more frequencies as well as could implemented in any growth stage of plant and finally could speed up breeding programs. Here in, the ISSR marker system was applied for evaluation of genetic variability of sumac genotypes as well. Paralleled with findings of Sutyemez et al. [17] remarkable polymorphism was detected through fingerprinting profile. Considering value of 0.5 as maximum value of PIC for dominant markers [27], the calculated PICs as indicator of primer power in germplasm screening showed suitability of applied ISSR primers in sumac. In the following, considering phytochemically identified sumac genotypes as superior genotypes (V6, V10, D10, D14, A1, A14, K3, K15, N10, and N11), the supervised machine learning analysis was done to detect which ISSR loci as features could achieve predefined groups. Although, similar with previous studies [20,21] the capability of feature selection algorithms was varied but they could effectively classify inspected sumac genotypes. In this way, 13 out of 36 implemented feature selection algorithms had high efficacy (more than 85 %) and identified ISSR loci (U823)L1, (U835)L1, (U801)L1, (U816)L2, (U816), L4(U835)L4, (U854)L1, and (U835)L9 as promising genomic regions (features) which playing key role in recognition of two sumac's groups. Such ISSR loci as functional markers could shorten schedule of phytochemical studies of sumac through germplasm screening in off-season.

## 5 Conclusion

There is a predominant genetic variability in sumac habitats from northwest of Iran. Such variabilities encourage selection of phytochemically enriched genotypes not only for germplasm conservation but also for achieving food security. Malic acid as one of phytochemical constituent of sumac's fruit was identified as a distinguishing agent which play key role in differentiation of sumac habitats from each other. In practice, it is important to select phytochemically superior sumac genotypes in early growth stages as well as off-season. This subject is accessible by implementation of ISSR marker data through machine learning and the present identified features (selected ISSRs) could be applied in sumac breeding program using marker-assisted selection.

## CRediT authorship contribution statement

**Hamid Hatami Maleki:** Writing – review & editing, Writing – original draft, Visualization, Software, Methodology, Investigation, Formal analysis, Data curation. **Reza Darvishzadeh:** Writing – review & editing, Supervision, Resources, Project administration, Investigation, Funding acquisition, Data curation, Conceptualization. **Ahmad Alijanpour:** Resources, Funding acquisition, Conceptualization. **Yousef Seyfari:** Writing – review & editing, Software, Formal analysis, Data curation.

## Data availability

Data will be made available on request.

## Funding

This research did not receive any specific grant from funding agencies in the public, commercial, or not-for-Profit sectors.

## Declaration of competing interest

The authors declare that they have no known competing financial interests or personal relationships that could have appeared to influence the work reported in this paper.
